# Factors that influence the Na/K-ATPase signaling and function

**DOI:** 10.3389/fphar.2025.1639859

**Published:** 2025-07-29

**Authors:** Yingnyu Gao, Yunhui Xu, Fang Bai, Raghav Puri, Jiang Tian, Jiang Liu

**Affiliations:** ^1^ Marshall Institute for Interdisciplinary Research (MIIR), Marshall University, Huntington, WV, United States; ^2^Bureau of Public Health, Office of Laboratory Services, West Virginia Department of Health, Big Chimney, WV, United States; ^3^Department of Biomedical Sciences, School of Medicine, Marshall University, Huntington, WV, United States; ^4^Department of Biomedical Engineering, Marshall University, Huntington, WV, United States

**Keywords:** Na/K-ATPase, cardiotonic steroids, signaling, ROS, pharmacological, implications

## Abstract

The transmembrane Na/K-ATPase is located in the plasma membrane of all mammalian cells. It utilizes energy from ATP hydrolysis to execute its pumping function and interacts with other proteins and/or kinase molecules to execute its signaling function. Digoxin, one of the earliest identified cardiotonic steroids (CTS) that specifically binds to the Na/K-ATPase, has been widely prescribed to manage patients with cardiovascular disease (CVD) and heart failure (HF) for over 200 years. Elevated plasma levels of CTS have been observed in patients with hypertension, chronic kidney disease (CKD), CVD, and congestive HF. After extensive research efforts spanning decades, there remain unresolved disagreements regarding the various mechanisms underlying the Na/K-ATPase signaling functions. This article examines the known and controversial mechanisms that initiate the Na/K-ATPase signaling functions and their related regulatory mechanisms.

## The Na/K-ATPase and cardiotonic steroids (CTS)

The P-type ATP-hydrolyzing enzyme Na/K-ATPase (EC 3.6.3.9) was first discovered as an energy-transducing primary ion-transporter (Skou, 1957), presented in all cell types tested. Unlike other P-type ATPases, Na/K-ATPase binds to a specific group of chemicals called cardiotonic steroids (CTS, also known as digitalis-like substances) that were recognized as a new class of endogenous steroid hormones (Schoner, 2002; Schoner et al., 2003). CTS has two structurally distinct groups: cardenolides (like ouabain and digoxin) and bufadienolides (like marinobufagenin MBG and telecinobufagin TCB). Digoxin has been highly prescribed to manage CVD and HF patients for over 200 years (Pavlovic, 2014). Elevated plasma CTS has been observed in patients with hypertension, CVD, CKD, CKD-related cardiomyopathy, and congestive HF, in which different Na/K-ATPase signaling functions were involved (Kelly and Smith, 1993; Bagrov et al., 1998; Kiselyov et al., 1999; Kaplan, 2002; Komiyama et al., 2005; Liu, 2005; Kolmakova et al., 2011; Bai et al., 2013; Pavlovic, 2014; Kennedy et al., 2015; Pavlovic, 2020).

### The history and progression of the Na/K-ATPase and CTS

A recent seminal retrospective review by Dr. Blaustein and Dr. Hamlyn discussed the history of the findings of different kinds of CTS (including endogenous ouabain), the mechanisms of their physiological and pathophysiological effects in cell models, animal models, and humans, the Na/K-ATPase-initiated intracellular signaling function by CTS, and some unresolved issues and possible solutions (Blaustein and Hamlyn, 2024). Another seminal review by Christian Staehr et al. focused on the potential implication of Na/K-ATPase-dependent intracellular signaling pathways in severe vascular disorders such as ischemic stroke, familial migraine, and arterial hypertension (Staehr et al., 2023).

## The signaling functions of the Na/K-ATPase

The Na/K-ATPase is highly sensitive to inhibition by CTS, which has been used for centuries to treat CVD, congestive HF and arrhythmias. The amino acids constituting the ouabain-binding site are highly conserved across the evolutionary spectrum to execute important biological functions that define the biological significance of the “receptor function” of the Na/K-ATPase and its regulation by potential endogenous CTS-like compounds (Lingrel, 2010), in which CTS binds to Na/K-ATPase at E2P state (Kanai et al., 2021). Furthermore, in the different kinds of CTS tested, there are kinetics and/or thermodynamic differences due to the type of sugar and lactone ring present in the steroid structure (Azalim-Neto et al., 2024), suggesting different possible Na/K-ATPase signaling pathways, binding properties, and functionalities.

The Na/K-ATPase has been extensively studied for its ion-pumping function in the kidney and heart in the early days. Later on, the revolutionary discovery that the NKA is also a hormone receptor and signaling molecule is essential for understanding the full impact of the NKA on physiology and pathophysiology (Blaustein and Hamlyn, 2024). The Na/K-ATPase has also been recognized as a receptor, signal transducer, signaling protein, and scaffolding protein through multiple protein-protein interactions in the past 3 decades (Xie and Askari, 2002; Xie and Cai, 2003; Pierre and Xie, 2006; Aperia, 2007; Bagrov et al., 2009; Li and Xie, 2009; Liu and Xie, 2010; Yan and Shapiro, 2016). Initially, it was found that CTS mediates signal transduction through the protein-protein interactions between Na/K-ATPase and other skeletal and/or kinase proteins. The signaling function of Na/K-ATPase has also been implicated in a range of clinical disorders, underscoring its broad relevance and potential for further research. These clinical disorders include, but are not limited to, cancer, CKD, CVD, and uremic cardiomyopathy. The potential for further research in this area is vast, offering many opportunities for discovery and advancement (Li et al., 2011; Yan and Shapiro, 2016). Over the past 30 years, the concept that the Na/K-ATPase functions as a signaling transducer has evolved significantly and is involved in many regulatory functions (Aydemir-Koksoy et al., 2001; Xie and Askari, 2002; Aizman and Aperia, 2003; Xie, 2003; Xie and Cai, 2003; Schoner and Scheiner-Bobis, 2005; Pierre and Xie, 2006; Schoner and Scheiner-Bobis, 2007; Bagrov et al., 2009; Liu and Xie, 2010; Liu et al., 2011; Aperia, 2012; Godinho et al., 2017; Wang and Shapiro, 2019; Wang et al., 2020; Liu et al., 2021; Maxwell et al., 2021; Horesh et al., 2024).

The Na/K-ATPase signaling function and activity are also linked to increased reactive oxygen species (ROS), in which CTS increases ROS generation, and increases in ROS can also initiate the Na/K-ATPase signal cascades in a feed-forward manner to form a Na/K-ATPase/ROS amplification loop (Yan et al., 2013; Yan and Shapiro, 2016; Liu et al., 2017; Liu et al., 2018). This mechanism has demonstrated significance in oxidative stress-related disease states, including obesity, atherosclerosis, heart failure, uremic cardiomyopathy, neurodegenerative disorders, hypertension, and other conditions rooted in ROS (Bundgaard and Kjeldsen, 1996; Srikanthan et al., 2016; Liu et al., 2018; Pratt et al., 2018; Liu et al., 2020; Staehr et al., 2022).

Ouabain is the most studied CTS. The binding of ouabain to the Na/K-ATPase α1 subunit causes increase in ROS, which can initiate the Na/K-ATPase signaling pathways that lead to increases in oxidative modification of the Na/K-ATPase α1 subunit, intracellular calcium concentration, and other effects. Receptors, signaling molecules, cytosolic proteins, and membrane structural proteins can interact with the Na/K-ATPase α1 subunit through multiple structural binding motifs found in the Na/K-ATPase α1 subunit (Sweadner and Donnet, 2001; Xie and Cai, 2003; Morth et al., 2007; Reinhard et al., 2013; Fedosova et al., 2021). These include but are not limited to c-Src, epidermal growth factor receptor (EGFR), phospholipase C (PLC), phosphoinositide 3-kinases (PI3K), inositol trisphosphate receptor (IP3Rs), ankyrin, adducin, and caveolin-1. The Na/K-ATPase signaling pathways have been demonstrated in different types of cells and animal models (Ferrandi et al., 2004; Periyasamy et al., 2005; Ferrandi et al., 2006; Kennedy, et al., 2006). Furthermore, the Na/K-ATPase α1/Src regulatory function of metabolic capacity is involved in metabolic diseases (Kutz et al., 2021; Staehr and Matchkov, 2021). Human induced pluripotent stem cells (hiPSC) expressing an Src-signaling null mutant (A420P) provide genetic evidence for the role of Na/K-ATPase α1/Src in the tonic stimulation of basal mitochondrial metabolism and ROS production in human cardiac myocytes (Cai, Pessoa, et al.). Using the porcine LLC-PK1 cells, incubation with 100 nM ouabain decreased ouabain-sensitive ^86^Rb uptake without significant impact on total enzyme activity, suggesting internalization of the Na/K-ATPase (Liu et al., 2002), which was further confirmed by the decreases of membrane-bound Na/K-ATPase using the biotinylation assay (Liu et al., 2004). The Na/K-ATPase trafficking occurs via a clathrin-dependent pathway by activating PI3K and Src (Liu and Xie, 2010). However, ouabain did not trigger significant Na/K-ATPase internalization in MDCK cells (Liu et al., 2002). On the other hand, in human endothelial cells, ouabain decreased rather than increased endocytosis of 3-(4,5-Dimethylthiazol-2-yl)-2,5-diphenyltetrazolium bromide (MTT) in human endothelial cells (Trevisi et al., 2006).

### The evolving functionalities of the Na/K-ATPase and CTS

Other than affecting the well-defined effects of the Na/K-ATPase in the modulation of cardiac and renal functions, studies have also demonstrated that the Na/K-ATPase has other emerging functions in many ways. For example, one newly synthesized CTS γ-Benzylidene Digoxin 8 (BD-8) is a promising immunomodulatory molecule, and another newly synthesized CTS 21-benzylidene digoxin (21-BD), but not ouabain, causes changes in cholesterol and phospholipid content (Silva et al., 2017; Silva et al., 2021; Silva et al., 2022), further suggesting possible different roles of CTS variants in signaling and cellular functions. Interestingly, CTS has different functions. For example, ouabain also affects neuroinflammation, acute onset of neurological symptoms, and neurological disorders (Holm and Lykke-Hartmann, 2016; Leite et al., 2022), enhances renal cyst growth in a slowly progressive mouse model of autosomal dominant polycystic kidney disease (ADPKD) (Trant et al., 2023), and the progression of ADPKD (Venugopal and Blanco, 2016), sperm energetic defects (Numata et al., 2022), pro-cytogenic effects in ADPKD (Venugopal and Blanco, 2017), as well as thymidine phosphorylase-mediated SARS-CoV-2 spike protein enhanced thrombosis in K18-hACE2TG mice and platelet GPCR signaling function in thrombosis (Li et al., 2024; Roytenberg et al., 2024). Even more interestingly, in neurons, impairment of Na/K-ATPase activity and signaling causes neuronal dysfunction due to affected Ca^2+^ signaling, and proper regulation of Na/K-ATPase activity and signaling is beneficial to brain function (Kinoshita et al., 2022). The normal Na/K-ATPase function is essential for neuronal function, including neurotransmitter release, electrical signaling, maintaining cell volume, and secondary transcellular transport. The Na/K-ATPase is crucial for neuronal function, consuming a significant portion of the brain’s energy to maintain cellular processes. About 60%–90% of Alzheimer’s disease (AD) patients exhibited reduced brain Na/K-ATPase activity and vascular dysfunction, further highlighting the potential role of Na/K-ATPase in AD (Zhang J. et al., 2022). This reduced brain Na/K-ATPase activity may be linked to the interaction of Na/K-ATPase with amyloid-beta (Aβ), a key protein implicated in AD pathology (Petrushanko et al., 2022). Specifically, Aβ oligomers, known as amylospheroids (ASPD), bind to the Na/K-ATPase α3 subunit in neurons, leading to presynaptic calcium overload and neuronal death (Ohnishi et al., 2015). These findings indicate a vast diversity of the possible Na/K-ATPase signaling functions in different studied subjects via different Na/K-ATPase subunits, ion homeostasis, and signaling partners. Furthermore, β-adrenergic regulation of the cardiac Na/K-ATPase mediated by oxidative signaling (Galougahi et al., 2012), and Src represents a key intermediate and novel therapeutic target in the pathophysiology of cerebral ischemia, where it appears to regulate neuronal damage by influencing VEGF-mediated vascular permeability (Paul et al., 2001).

**FIGURE 1:**
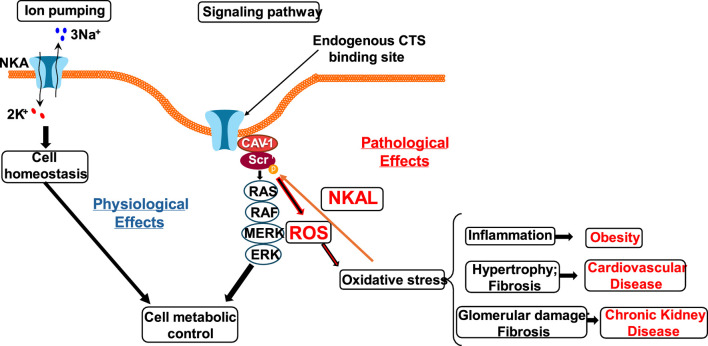
Schematic depicting the proposed disease pathogenesis progression with central oxidant stress production through the Na/K-ATPase signaling. Reactive oxygen species (ROS) lead to Na/K-ATPase conformational changes that activate c-Src and initiate downstream signaling cascades. This activation leads to ROS overproduction and oxidative stress, contributing to the development of obesity, cardiovascular, and renal diseases.

The Na/K-ATPase-dependent Src kinase activation is the key mechanisms responsible for elevation of cerebrovascular tone after reperfusion and the initiation of the Src kinase signaling pathway that sensitizes the contractile machinery to intracellular Ca^2+^ resulting in hypercontractility of vascular smooth muscle cells and, thus, elevated cerebrovascular tone that can contribute to impaired reperfusion after stroke, the potential implication of Na/K-ATPase-dependent intracellular signaling pathways in severe vascular disorders such as ischemic stroke, familial migraine, and arterial hypertension (Guldbrandsen et al., 2021; Staehr et al., 2023). Furthermore, inhibition of the Na/K-ATPase-dependent Src kinase signaling with pNaKtide prevented excessive vasoconstriction and disturbances in neurovascular coupling in a NKA α2+/G301R mouse model. pNaKtide had only a minor hypotensive effect, similar in both genotypes. The results demonstrate a novel treatment target to normalize cerebral perfusion in FHM2 (Staehr et al., 2025). The underlying mechanisms integrate cerebral edema, oxidative stress, and Na/K-ATPase-related c-Src signaling. The Na/K-ATPase signaling function and activity are also linked to increased reactive oxygen species (ROS) in a bidirectional way, in which CTS increases ROS generation, and increases in ROS also initiate the Na/K-ATPase signal cascades in a feed-forward manner (Yan et al., 2013; Yan et al., 2016; Liu et al., 2017; Liu et al., 2018). This Na/K-ATPase/ROS amplification loop has demonstrated the significance in oxidative stress-related disease states, including obesity, atherosclerosis, heart failure, uremic cardiomyopathy, neurodegenerative disorders, hypertension, and other conditions rooted in ROS (Figure 1).

## The proposed models of the Na/K-ATPase α1-Src interaction and signaling

The formation of the Na/K-ATPase α1-Src complex, a key step in the primary Na/K-ATPase signaling function, remains a topic of debate. There were evidences suggesting that Na/K-ATPase directly interacts with Src and other proteins (like caveolin-1) to form a functional receptor complex in live cells, as demonstrated by various available technologies at different times, as well as that the Src activation is through an ATP/ADP ratio change or ATP-sparring function, but not through the direct interaction with the Na/K-ATPase α1 subunit. Even though there is uncertainty about the proposed different signaling modeling, the undefined mechanisms might affect the proposed capabilities and functions of c-Src activation. An organized functional signaling receptor complex might be more effective and controlled than any “random” reaction. These ongoing debates provide a comprehensive understanding of the primary Na/K-ATPase-Src signaling function, paving the way for further research and potential therapeutic applications (Liu et al., 2003; Wang et al., 2004; Kotova et al., 2006; Liang et al., 2006; Tian et al., 2006; Morth et al., 2009; Yatime et al., 2009; Weigand et al., 2012; Yosef et al., 2016; Nie et al., 2020).

Three different CTS-stimulated Src kinase activation “working” models have been proposed to explain the mechanisms underlying the activation of the Na/K-ATPase signaling function.

(1) The First Model is the direct interaction of the Na/K-ATPase α1 subunit with c-Src, which forms a functional Na/K-ATPase/c-Src signaling receptor complex in caveolae. In this Model, the Na/K-ATPase α1 subunit binds to c-Src through two pairs of domain binding (Na/K-ATPase α1 CD2 segment with c-Src SH2 domain, and Na/K-ATPase α1 ND1 segment with c-Src kinase domain) under resting conditions, and ouabain stimulates the release of c-Src KD from α1 ND1 segment that leads to c-Src kinase domain Tyr-418 phosphorylation (Wang et al., 2004; Tian et al., 2006). The binding status was affected by the changes of Na/K-ATPase E1/E2 conformation change and intracellular sodium and potassium concentration (Ye et al., 2011) This was further supported that expression of Na/K-ATPase α1 mutants (rat α1 mutants I279A and F286A) with defective in E1/E2 transition altered both basal and stimuli-induced Src regulation (Ye et al., 2013). Ouabain not only regulated the interaction in a time- and dose-dependent manner and stimulated tyrosine phosphorylation of caveolin-1 in LLC-PK1 cells but also induced the formation of a Na/K-ATPase-Src-caveolin complex and increased tyrosine phosphorylation of proteins isolated from caveolae. Furthermore, depletion of either cholesterol by methyl beta-cyclodextrin or caveolin-1 by siRNA significantly reduced the caveolar Na/K-ATPase and Src, which abolished ouabain-induced recruitment of Src to the Na/K-ATPase signaling complex (Wang et al., 2004; Tian et al., 2006). In this Model, the Na/K-ATPase α1 subunit provides the ligand binding sites, the α1-associated c-Src provides the kinase moiety, and the caveolin-1 functions as an anchor to enrich the signaling partners in the caveolae structure. This Model was tested based on cell-free and live cells with different manipulations of the partner proteins (the α1 subunit, c-Src, and caveolin-1).
(2) The Second Model proposed that c-Src only transiently interacts with a protein complex formed between the Na/K-ATPase α1 and caveolin-1. To evaluate if there are direct interactions amongst Na/K-ATPase, Src, and caveolin-1, purified recombinant Na/K-ATPase (human α1β1FXYD1 or porcine α1D369Nβ1FXYD1) and purified human Src kinase and human caveolin-1 in native membrane vesicles isolated from rabbit kidney were applied. It showed no stable direct interactions between Na/K-ATPase and purified Src kinase, but a direct interaction between purified human Na/K-ATPase and human caveolin-1 (Yosef et al., 2016). This model was established in a cell-free system, with over-expressed and purified partner proteins, isolated plasmalemma caveolae, and right-side-out vesicles from rabbit kidney outer medulla. The first and second models agree that caveolin-1, by binding to the Na/K-ATPase α1 subunit, functions as an anchoring protein to concentrate the “signaling” α1 subunit and its signaling partners in the caveolae structure. Another common characteristic of these two models is that c-Src activation is a proximal step in the Na/K-ATPase signaling initiated by either stable or transient interaction between the α1 subunit and c-Src. In the second Model, the Tyr-418 in purified c-Src was already phosphorylated in the isolation/purification process before binding experiments, which might leave only one pair of possible domain binding (i.e., Na/K-ATPase α1 CD2 domain with c-Src SH2 domain) under the experiment condition that might weaken the total binding force between the α1 subunit and c-Src proposed in the first Model.
(3) The Third Model proposed that c-Src activation is primarily a consequence of an ATP-sparing effect induced by the Na/K-ATPase inhibitors (ouabain, vanadate, and oligomycin) and energy status (ATP/ADP ratio). Ouabain-induced activation of PI3K/Akt and MAPK/ERK1/2 was independent of Src (Liu et al., 2007; Wu et al., 2013; Gable et al., 2014) but dependent on the ATP/ADP ratio induced by Na/K-ATPase inhibitors (digoxin, vanadate, Na+, K+, ATP, and ADP) in a dose-dependent manner, without protein-protein interaction between the Na/K-ATPase α1 subunit and c-Src (Weigand et al., 2012).

Although there are controversies regarding the direct or indirect interaction between the Na/K-ATPase and Src kinase, the activation of Src kinase by phosphorylation is a well-established response to sub-micromolar concentrations of ouabain that was demonstrated in all three proposed models. Studies suggest that the Na/K-ATPase-associated Src kinase activated by ouabain (Haas et al., 2000; Liang et al., 2006; Tian et al., 2006; Li et al., 2009; Lai et al., 2013; Ye et al., 2013; Banerjee et al., 2015). This signaling microdomain model disagreed with other studies suggesting that ouabain-induced Src kinase activation is a result of the ATP-sparing effect of the Na/K-ATPase inhibitors (Weigand et al., 2012; Gable et al., 2014). However, in skeletal muscle, sub-micromolar and micromolar concentrations of ouabain do not affect the global intracellular ATP/ADP ratio, but with significant phosphorylation and activation of Src kinase (Kotova et al., 2006). Nevertheless, these studies addressed the global ATP/ADP ratio, and there is a possibility for spatially restricted changes in the ouabain concentrations. Accordingly, the Na/K-ATPase-dependent Src kinase activity is maintained in cells expressing a non-pumping mutant of the rat α1 isoform (Liang et al., 2006). The involvement of the Na/K-ATPase in the signaling cascade does not exclude a role for its ion-pumping function in ouabain-induced effects. Moreover, since intracellular Na^+^ ions regulate the conformation change of the Na/K-ATPase, it is possible that changes in intracellular Na^+^ concentration could also regulate the formation of the Na/K-ATPase/Src signaling complex, and thus cellular Src activity (Li et al., 2009). This Na/K-ATPase-dependent Src kinase signaling can modulate arterial contraction and blood pressure, as discussed below.

Notably, a common characteristic in these three models is that the E2(P) conformational state of the Na/K-ATPase is favored and stabilized by the Na/K-ATPase inhibitors (ouabain, vanadate, oligomycin), and energy status (ATP/ADP ratio). Though the dynamic conformational changes can affect the formation of the signaling complex, c-Src activation is one of the most proximal steps in the Na/K-ATPase signaling since the Na/K-ATPase itself lacks tyrosine kinase activity. The discrepancies amongst these models could be attributed to the different experimental designs and conditions, since the interactions of the Na/K-ATPase α1 subunit with c-Src and/or caveolin-1 might also require other protein(s) that are not present in some experimental conditions, as suggested in the Second Model (Yosef et al., 2016).

Under the native conditions, Blue Native-PAGE, Blue Native-PAGE/SDS-PAGE 2D, and Mass-Spec analyses demonstrated the co-existence of the Na/K-ATPase α1 subunit and c-Src in the same protein complex and a direct interaction between the Na/K-ATPase α1 subunit and c-Src (Nie et al., 2020). These observations were further supported by comparisons of DTT-cleavable and DTT-non-cleavable cysteine-cysteine (SH-SH) crosslinked samples, which demonstrated that depletion of Src kinase family members (c-Src, Yes, and Fyn) or caveolin-1 reduced the interactions of the Na/K-ATPase α1 subunit with other proteins, but depletion of caveolin-1 did not affect the interaction of c-Src with the Na/K-ATPase α1 subunit. The data showed direct interactions between the Na/K-ATPase α1 subunit and c-Src and between the Na/K-ATPase α1 subunit and caveolin-1. However, the data argued about the interaction between c-Src and caveolin-1 under the native condition. Furthermore, the data also indicated the existence of different protein complexes containing the Na/K-ATPase α1 subunit and c-Src, which might have different signaling functions (Nie et al., 2020). Therefore, further studies are expected to determine whether the Na/K-ATPase-Src interaction and signaling function under different conditions.

## The caveolae and caveolins in the Na/K-ATPase signaling

The caveolae are microdomain rafts containing caveolins that are capable of assembling and regulating signaling complexes through association with a variety of membrane receptors and play pleiotropic roles in the regulation of cellular functions (Anderson, 1998; Drab et al., 2001; Razani et al., 2002; Gratton et al., 2004; Wang et al., 2004), such as hypertrophic signaling (Ferrandi et al., 2004; Ferrandi et al., 2006). In caveolae isolated from cardiomyocytes, cardiac ventricles, kidney cell lines (porcine renal proximal tubular LLC-PK1 cell, human embryo HEK-293 cell, and porcine kidney outer medulla), the isolated caveolae (by detergent-free procedures) contain Src, EGFR, ERK1/2, Na/K-ATPase α1/α2, and caveolin-3 (Song et al., 1996; Liu et al., 2003; Wang et al., 2004; Cui and Xie, 2017). Furthermore, in cardiac caveolae isolated from ouabain-treated contracting rat hearts, there were significant increases in phosphorylated ERK1/2 and recruitment of Src and Na/K-ATPase α2 to caveolae (Liu et al., 2003).

The primary sequence of caveolin-1 contains a central hydrophobic domain (residues 102–134) that anchors to membranes, an oligomerization domain (residues 61–101), and a scaffolding domain (residues 82–101) (Schlegel and Lisanti, 2001). Interaction between the oligomerization domains and the C-terminal domains results in the formation of high molecular oligomers containing about 14–16 caveolins, which is important for the scaffolding function of caveolins. The interaction of the caveolin scaffolding domain with putative caveolin-binding motifs in many signaling proteins, such as Src, EGFR, and Ras, concentrates these proteins in caveolae (Schlegel and Lisanti, 2001). The Na/K-ATPase expression regulates caveolin-1 endocytic trafficking and stabilizes the caveolin-1 plasma membrane pool, which was independent of the Na/K-ATPase pumping function but depends on the interaction between Na/K-ATPase and caveolin-1 (Cai et al., 2008a). Moreover, knockdown of the Na/K-ATPase increases basal levels of active Src and stimulates endocytosis of caveolin-1 from the plasma membrane (Cai et al., 2008b), and the caveolar Na/K-ATPase interacts with caveolin-1 and Src to form a signaling complex, which was induced by ouabain-stimulated tyrosine phosphorylation of caveolin-1 (Wang et al., 2004). Other than Src, the Na/K-ATPase α1 also interacts with other partners, including phosphoinositide 3-kinase (PI3K) and caveolin-1, and is involved in regulating the PI3K/Akt pathway and the formation of caveolae (Cai et al., 2008b; Tian et al., 2009). In the meantime, Na/K-ATPase and CTS-induced signal transduction of protein kinases and calcium-signaling microdomain in the regulation of transporter trafficking (Liu and Xie, 2010). While depletion of caveolae increases the pumping function of Na/K-ATPase, it suppresses CTS-induced signal transduction, cell growth, and collagen production in cardiac fibroblasts (Quintas et al., 2010).

Moreover, over half of the plasma membrane Na/K-ATPase in LLC-PK1 cells performs cellular functions other than ion-pumping. Like the pumping pump, this “non-pumping” pool of the Na/K-ATPase binds ouabain. Depletion of either cholesterol or caveolin-1 moves the “non-pumping” Na/K-ATPase into the pumping pool. Graded knockdown of the Na/K-ATPase α1 subunit eventually results in losing this “non-pumping” pool while preserving the pumping pool. A loss of the “non-pumping” pool is associated with a loss of receptor function, as evidenced by the failure of ouabain to induce the activation of Src and/or ERK. These findings suggest that a substantial amount of surface-expressed Na/K-ATPase, at least in some types of cells, may function as non-canonical ouabain-binding receptors (Liang et al., 2007). The caveolar Na/K-ATPase represents the signaling pool of the Na/K-ATPase that interacts with Src and transmits the ouabain signals (Wang et al., 2004). Ouabain interacts with the Na/K-ATPase α1 that resides within the caveolar domain. This interaction selectively recruits signal-transducing proteins to this microdomain, activating their signaling functions, which is necessary to initiate the proliferative cascade (Liu et al., 2004). This transducing function occurred at ouabain concentrations that do not perturb cytoplasmic ion content and requires specific localization of the Na/K-ATPase to caveolae (Allen et al., 2003). The ouabain-induced recruitments of Src and Na/K-ATPase α-subunit to caveolae are involved in the positive inotropic effect of ouabain (Liu et al., 2003) and play differential roles of caveolin-1 in ouabain-induced Na/K-ATPase cardiac signaling and contractility (Bai et al., 2016). In caveolin-1 knockdown LLC-PK1 cells, ouabain failed to downregulate NHE3 mRNA expression and NHE3 promoter activity (Oweis et al., 2006). The link of Na/K-ATPase to ERK1/2 and intracellular Ca^2+^ was organized within cardiac caveolae microdomains, and ouabain-induced recruitments of Src and Na/K-ATPase α2 to caveolae were involved in the positive inotropic effect of ouabain (Liu et al., 2003). While both PLC-γ1 and IP3 receptors (isoforms 2 and 3) were coenriched with the Na/K-ATPase, caveolin-1, and Src, pulldown assays revealed that the central loop of the Na/K-ATPase α1 subunit interacts with PLC-γ1, and the N-terminus of the Na/K-ATPase α1 subunit binds IP3R2 and IP3R3, suggesting that the signaling Na/K-ATPase may tether PLC-γ1 and IP3 receptors to form a Ca^2+^-regulatory complex that requires c-Src (Yuan et al., 2005).

The gain of the caveolin binding motif (CBM) in the Na/K-ATPase α1 subunit enables the Na/K-ATPase to function as a dual-functional protein for stem cell differentiation and organogenesis in multicellular organisms that are involved in the genetic regulation of myogenesis through Wnt/β-catenin signaling (Wang et al., 2020; Huang et al., 2022). The conserved N-terminal CBM of the Na/K-ATPase α1 allows Na/K-ATPase/caveolin-1 interaction and influences Na/K-ATPase signaling and caveolar distribution that is critical for animal development, ontogenesis, and lineage-specific differentiation of human induced pluripotent stem cells (hiPSCs). An altered CBM in hiPSC-derived adipocytes (iAdi-mCBM) and in mice (mCBM) showed impaired glycolysis and decreased ATP synthesis-coupled respiration in iAdi-mCBM with extensive remodeling of the extracellular matrix (ECM) and heightened TGF-β signaling. The Na/K-ATPase CBM signaling integrity is required for adequate control of TGF-β signaling and ECM stiffness during adipogenesis (Huang et al., 2024). A genetic approach to alter the Na/K-ATPase α1 CBM in hiPSC-derived adipocytes and mice indicated that the Na/K-ATPase CBM regulates adipogenesis in the adipocytes and reduces fat with increased extracellular matrix production and inflammation in mice. RNA-seq analysis and pharmacological interventions in human hiPSC-derived adipocytes revealed that the TGF-β signal, rather than Na/K-ATPase-mediated ion transport, is a key mediator of Na/K-ATPase-induced regulation of adipogenesis (Huang et al., 2024).

## The clathrin in Na/K-ATPase signaling and renal function

Ouabain-induced endocytosis of plasmalemmal Na/K-ATPase in LLC-PK1 cells by a clathrin-dependent but non-species-specific mechanism, which also involved GRP78/BiP (Liu et al., 2004a; Kesiry and Liu, 2005; Liu et al., 2006; Liu et al., 2005; Yan et al., 2012). Ouabain activates the basolateral Na/K-ATPase-PI3K signaling pathway, stimulates NHE3 trafficking by the basolateral Na/K-ATPase signaling complex, which was abolished by cholesterol depletion, Src inhibition, and intracellular Ca^2+^ chelator BAPTA-AM treatment (Kaunitz, 2006; Oweis et al., 2006; Blaustein et al., 2007; Cai et al., 2008). Ouabain, at low doses without changing the concentration of intracellular Na^+^, significantly reduced NHE3 activity, NHE3 protein content, and mRNA expression. Inhibition of c-Src or PI3K with PP2 or wortmannin, respectively, abolished ouabain-induced downregulation of NHE3 activity and mRNA expression. It has been shown that binding of ouabain to Na/K-ATPase activates Src/EGFR signaling to initiate multiple signal pathways that regulate cell growth. In porcine LLC-PK1 cells, ouabain decreased ouabain-sensitive ^86^Rb uptake without significant impact on total enzyme activity, suggesting internalization of the Na/K-ATPase (Liu et al., 2002; Liu et al., 2004), and the Na/K-ATPase trafficking occurs via a clathrin-dependent pathway by activation of PI3K and Src (Liu and Xie, 2010). However, ouabain did not trigger Na/K-ATPase internalization in Madin-Darby canine kidney (MDCK) cells (Liu et al., 2002; Akimova et al., 2008), and ouabain decreased, rather than increased, endocytosis (Akimova et al., 2006; Trevisi et al., 2006). In LLC-PK1, but not MDCK cells, low concentrations of ouabain decreased ^86^Rb uptake and transcellular ^22^Na flux profoundly in a time and dose-dependent manner. The binding of CTS marinobufagenin (MBG) and deproteinated extract of serum derived from patients with chronic renal failure by proximal (but not distal) tubular cells results in internalization of Na/K-ATPase with the net effect of amplifying inhibition of the Na/K-ATPase activity (Liu et al., 2002). Furthermore, *in vivo* and *in vitro* studies with Dahl salt-sensitive (S) and salt-resistant (R) rats and their primary culture of renal proximal tubules isolated from Dahl S and R rats, high salt diet or ouabain treatment stimulated Na/K-ATPase/c-Src signaling and internalization of Na/K-ATPase and NHE3 in the Dahl R rats, but not in the Dahl S rats, suggesting that impairment of Na/K-ATPase signaling and consequent regulation of Na/K-ATPase and NHE3 in renal proximal tubule that contribute to salt-induced hypertension in the Dahl S rat, and increased basal level of renal Na/K-ATPase-dependent redox signaling may be responsible for the development of salt-sensitive hypertension in polygenic obese TALLYHO/JngJ mice (Liu et al., 2011; Yan et al., 2019). Moreover, the extracellular renal interstitial guanosine cyclic 3′,5′-monophosphate (cGMP) inhibits renal proximal tubule Na^+^ reabsorption via Src activation through cGMP binding to the extracellular Na/K-ATPase α1 subunit on the basolateral membranes to inhibit Na^+^ transport. Molecular modeling, by using data from competitive binding studies and crosslinking studies, strongly suggested a potential cGMP docking site in the ouabain-binding pocket of Na/K-ATPase (Bulger and Griendling, 2023; Kemp et al., 2023).

## The Na/K-ATPase α1 expression and Na/K-ATPase signaling

The number of Na/K-ATPase in the plasma membrane of LLC-PK1 cells is about one million per cell, and about half reside in caveolae (Liang et al., 2007). In LLC-PK1 cells, graded knockdown of Na/K-ATPase α1 subunit decreases the plasma membrane pool of caveolin-1 and significantly reduces the number of caveolae on the cell surface. However, knockdown of Na/K-ATPase α1 subunit increases basal levels of active Src and stimulates endocytosis of caveolin-1 from the plasma membrane, indicating that the Na/K-ATPase regulates caveolin-1 endocytic trafficking and stabilizes the caveolin-1 plasma membrane pool (Cai et al., 2008b). The PY-17 cells were derived from porcine LLC-PK1 cells, and the expression of endogenous Na/K-ATPase α1 was reduced by about 90%. Both cell-free and cell-based assays indicate that the A420P mutation abolishes the Src regulatory function of Na/K-ATPase but has a normal ion-pumping function, indicating the Na/K-ATPase α1 possesses both pumping and signaling functions (Lai et al., 2013). On the other hand, graded knockdown of Na/K-ATPase in LLC-PK1 cells decreases the plasma membrane pool of caveolin-1, significantly reducing the number of caveolae on the cell surface. However, it increased basal levels of active Src and stimulated endocytosis of caveolin-1 from the plasma membrane, indicating the Na/K-ATPase also regulates caveolin-1 endocytic trafficking and stabilizes the caveolin-1 plasma membrane pool (Cai et al., 2008a). Furthermore, Na/K-ATPase α1 haploinsufficiency led to enhanced lipopolysaccharides (LPS)-stimulated NF-κB pathway, ROS signaling, and proinflammatory cytokines through Na/K-ATPase α1 interaction with TLR4 and Lyn, which leads to the suppression of LPS-induced macrophage proinflammatory signaling through Lyn (Zhang et al., 2022). Furthermore, knockdown of RPT Na/K-ATPase in cells and mice increased membrane NHE3 and Na^+^/HCO^3^−cotransporter (NBCe1A), decreased urine output and absolute Na + excretion driven by increased RPT Na + reabsorption and accompanied by elevated blood pressure. This re-absorptive phenotype was rescued upon crossing with RPT NHE3^−/−^ mice, confirming the importance of Na/K-ATPase/NHE3 coupling, indicating that Na/K-ATPase signaling is not only physiologically relevant but also functionally dominant over Na/K-ATPase ion-pumping function in the control of RPT reabsorption (Mukherji et al., 2023). Different structural CTS types could trigger different sets of Na/K-ATPase/effector interactions, resulting in biased signaling responses without compromising ion-pumping capacity (Xu et al., 2021), and the Na/K-ATPase α1–Src kinase complex plays a central role in regulating the renal inflammatory response induced by elevated CTS both *in vitro* and *in vivo* (Khalaf et al., 2019).

## The expansion of the NA/K-ATPase signaling

Beyond the above-discussed Na/K-ATPase-Src signaling pathways and functions, the Na/K-ATPase α1 subunit was also shown to interact with other proteins to initiate signaling function to affect other cellular and whole-body functions, such as diet-induced obesity and hyperlipidemia, potentiate a CD36/Na/K-ATPase–dependent inflammatory paracrine loop between proximal tubule cells and their associated macrophages, thereby facilitate the development of chronic inflammation and tubulointerstitial fibrosis, obesity, and cardiovascular diseases (Kennedy et al., 2013; Yan and Shapiro, 2016; Yoo et al., 2022). The Na/K-ATPase signaling functions might regulate many functions, such as that blockage of the Na/K-ATPase signaling-mediated oxidant amplification loop elongates red blood cell half-life and ameliorates uremic anemia induced by 5/6th PNx in C57BL/6 mice, suggesting the role of Na/K-ATPase signaling in the regulation of red blood cells (Liu et al., 2022). Evidence indicates that oxidative stress regulates the Na/K-ATPase enzymatic activity, signaling, and other functions. While a CTS-induced increase in ROS generation is an intermediate step in CTS-mediated Na/K-ATPase signaling, an increase in ROS alone also stimulates Na/K-ATPase signaling, salt-sensitivity, fibrosis, and RPT sodium reabsorption (Liu et al., 2012; Dial et al., 2014; Shah et al., 2016; Liu et al., 2017). Nevertheless, there are many more regulatory effects of the Na/K-ATPase signaling function that we cannot list here, and we expect more evidence to emerge with further studies since the expression of the Na/K-ATPase is found in all kinds of cells.

## The pharmacological implications of the Na/K-ATPase expression and signaling, CTS, and oxidative stress

Up to date, the dysregulation of these factors affects almost every pathophysiological condition studied in humans, animals, and cells. Clinical and animal studies have shown that reduced NKA α1 expression and activated NKA signaling function are significant risk factors for renal and cardiac dysfunctions (Norgaard et al., 1988; Semb et al., 1998; Ishino et al., 1999; Moseley et al., 2004; Liu et al., 2012), where CTS, NKA expression, and oxidative stress play critical roles (Haas et al., 2000; Liu et al., 2000; Xie and Askari, 2002; Aizman and Aperia, 2003; Miyakawa-Naito et al., 2003; Xie, 2003; Liu et al., 2004; Kaunitz, 2006; Kotova et al., 2006; Li et al., 2006; Pierre and Xie, 2006; Schoner and Scheiner-Bobis, 2007; Wansapura et al., 2010; Aperia, 2012; Yan et al., 2013; Pratt et al., 2018; Liu et al., 2020; Marck et al., 2021). The Na/K-ATPase expression and signaling, CTS, and oxidative stress are inextricably and functionally linked, as in the bidirectional cardiorenal and/or renocardiac syndromes. It has demonstrated that (1) “non-toxic” concentrations of ouabain (without significant inhibition of NKA activity) stimulated NKA signaling and cardiac hypertrophy that were linked to oxidative stress, but are independent of intracellular [Na^+^]_i_ and [Ca^2+^]_i_ (Xie et al., 1999; Liu et al., 2000); (2) Glucose oxidase-induced sustained low level of H_2_O_2_ stimulated [Ca^2+^]_i_-independent cardiac hypertrophy, NKA α1 trafficking, and NKA activity inhibition (Liu et al., 2006); (3) a NKA-Src signaling inhibitor, pNaKtide, attenuated PNx-stimulated NKA signaling, oxidative stress, and cardiac dysfunction (Liu et al., 2016); (4) Clinically, patients with HF or idiopathic dilated cardiomyopathy showed significantly reduced NKA α1 expression and activity (Norgaard, Bagger et al., 1988, Schmidt et al., 1993; Ishino et al., 1999; Moseley et al., 2004); and (5) a weighted gene co-expression network analysis (WGCNA) of published RNA-sequencing data (GEO141910) from a cohort of 366 heart-transplant patients and their donors, the expressions of NKA α1 and α2 are significantly correlated with left ventricular ejection fraction (LVEF) (Gao et al., 2022).

The apical sodium-glucose cotransporter 2 (SGLT2) is responsible for about 90% renal glucose reabsorption from the glomerular filtrate in the S1/S2 segment of renal proximal tubules (RPTs), driven by the Na^+^ gradient generated by the apical Na^+^/H^+^ exchanger isoform 3 (NHE3) and basolateral Na/K-ATPase. SGLT2 inhibitors (SGLT2i) not only have “on-target” mild glucose-lowering effects but also have profound “off-target” beneficial renal and cardiovascular effects in patients with CKD, CVD, and HF, through glucosuria, natriuresis, metabolic shifts, and reduction of oxidative stress, that are independent of hyperglycemia, sex, and insulin secretion/sensitivity. Clinical studies have shown that SGLT2i-induced beneficial effects in CKD and CVD/HF patients are independent of hyperglycemia, sex, and insulin secretion/sensitivity. The underlying mechanisms include, but are not limited to, lowering blood pressure and blood glucose, improving RPT function and cardiomyocyte calcium handling and myocardial energetics, normalizing oxidative stress, stimulating erythropoiesis and erythropoietin, and volume contraction to improve oxygen delivery, inducing autophagy, and reducing epicardial fat (Lambers Heerspink et al., 2013; Pessoa et al., 2014; Vallon, 2015; Tanaka et al., 2018; Mosenzon et al., 2019; Perkovic et al., 2019; Zelniker et al., 2019; Ghanim et al., 2020; Heerspink et al., 2020; Mazer et al., 2020; Santos et al., 2020; Vallon and Thomson, 2020; Almaimani et al., 2021; Borges-Júnior et al., 2021; Joshi et al., 2021; Vallon and Verma, 2021; Vallon and Verma, 2021).

These findings signify the roles of NKA expression, signaling, and oxidative stress in cardiorenal and renocardiac syndromes. Furthermore, clinical trials have demonstrated that SGLT2i are more effective in CKD and CVD/HF patients than healthy individuals. Therefore, further defining the role of these factors holds immense potential for managing CKD and CVD/HF, which are still largely untreatable clinical entities.

Since the broad effects of CTS and Na/K-ATPase signaling, it has been widely accepted that it is clinically significant in other possibilities other than regulating renal and cardiac function. Cinobufagin, a cardiotoxic bufadienolide steroid, has the potential to be further developed as a new drug against cancer. Cinobufagin significantly inhibited the growth of Skov3 ovarian cancer cells, triple-negative breast cancer metastasis, by regulating the Forkhead Box S1 (FOXS1) gene, the CCL2/CCR2 signaling, and FAK/STAT3 signaling (Dai et al., 2023; Zhu et al., 2024; Wang et al., 2025). More excitingly, in a proof-of-concept clinical trial, digoxin treatment (0.7–1.4 ng/mL serum level) reduced circulating tumor cell clusters in metastatic breast cancer, which are associated with disease progression and reduced survival in a variety of cancer types (Kurzeder et al., 2025). Clinic trials also demonstrated that rostafuroxin (a digitoxigenin derivative and a ouabain antagonist that selectively disrupts the binding to the cSrc-SH2 domain of mutant α-adducin and of the ouabain-activated NKA) has anti-hypertensive effects that are linked to genetic backgrounds (Lanzani et al., 2010; Citterio et al., 2021). In a randomized, placebo-controlled, double-blind study, using an anti-digoxin antibody Fab in women with severe preeclampsia with positive endogenous digitalis-like factors (EDLFs) status was associated with improved maternal and neonatal outcomes (Lam et al., 2013). Furthermore, in a rat model of preeclampsia, treatment with anti-MBG antibody was effective at normalizing blood pressure, kidney function, and fetal birth weights (Pantho et al., 2025), and in a rat partial nephrectomy model, treatment with anti-MBG antibody counteracts the Fli1-collagen-1 system, and reduces aortic fibrosis (Agalakova et al., 2024). Aldosterone antagonists (canrenone and canrenoate), exert both agonist and antagonist effects on the digitalis receptor site of NKA, can both stimulate and inhibit the NKA to affect blood pressure in human and animal models of volume expanded hypertension, and the Milan hypertensive rat models of hypertension (Grichois et al., 1986; Semplicini et al., 1995).

Src represents a key intermediate and novel therapeutic target in the pathophysiology of cerebral ischemia. Mice lacking pp60c-src are resistant to vascular endothelial growth factor (VEGF)-induced vascular permeability (VP) and show decreased infarct volumes after stroke, whereas mice deficient in pp59c-fyn, another Src family member, have normal VEGF-mediated VP and infarct size. Systemic application of a Src-inhibitor given up to 6 h following stroke suppressed VP, protecting wild-type mice from ischemia-induced brain damage without influencing VEGF expression (Paul et al., 2001). The administration of the Src-family tyrosine kinase inhibitor PP2 attenuated transient focal ischemia-induced increase of tyrosine phosphorylation of occludin in the isolated brain capillaries, which was coincident with an inhibition of blood-brain barrier (BBB) leakage and a decrease in infarct volume (Takenaga et al., 2009). Inhibition of p-Src by PP2 also ameliorated neuropathological changes and damaged neurological functions induced by hypoxic-ischemic injury in a Sprague-Dawley rat model (Qiu et al., 2021).

## Summary and perspective

The Na/K-ATPase (NKA) functions as an ion pump and a signaling molecule. It is sensitive to inhibition by CTS, which has implications for cardiovascular and kidney diseases. NKA signaling is linked to reactive oxygen species (ROS) production and metabolic regulation. The Na/K-ATPase α1-Src complex plays a crucial role in cellular signaling. Three main models explain the interaction between NKA and c-Src, with ongoing debates. Caveolae and caveolin proteins are involved in NKA-mediated signal transduction. The signaling function of NKA extends beyond ion transport, influencing conditions like hypertension, obesity, and Alzheimer’s disease. The crosslinking methods (Blue Native-PAGE, immunoblotting, and capillary immunoblotting) help map protein-protein interactions, including NKA’s interactions with c-Src and caveolin-1, which provide insights into NKA’s structural organization and its role in disease that might aid in drug development by identifying potential therapeutic targets. Furthermore, the direct interaction between NKA α1 and c-Src, and related oxidative stress, highlights its role in signaling and disease. It has been shown that under normal conditions, NKA α1 binds to and inhibits c-Src. Upon stimulation (e.g., by CTS), NKA undergoes conformational changes, releasing and activating c-Src; activated c-Src triggers downstream signaling pathways linked to cell growth, inflammation, and disease; dysregulation of this interaction is associated with cardiovascular disease, kidney disease, cancer, and other disease conditions. The present studies collectively highlight Na/K-ATPase’s dual function as an ion pump and a signaling hub. While much progress has been made, there are still unresolved questions, particularly regarding its exact signaling mechanisms and therapeutic applications. Despite recent advances in elucidating the possible mechanisms of the Na/K-ATPase-related signaling function, different proposed models depend on the different experimental settings, materials used, and explanations. Each proposed mechanism has its merits and should be further evaluated. Nevertheless, a deeper understanding of the Na/K-ATPase-related signaling function would advance our understanding of its effects on health and disease. Future research is needed to resolve controversies in its signaling mechanisms. Future studies will aim to resolve current controversies (e.g., the nature of the Na/K-ATPase/c-Src complex) and explore the broader implications of the Na/K-ATPase expression and signaling in cellular regulation and pathology, such as ROS production, cellular metabolism, the progression of diseases such as hypertension, obesity, cardiorenal or renocardiac syndrome, and neurodegenerative disorders, that will benefit future clinic therapeutic strategies.
